# *In vivo* spectroscopic evaluation of human tissue optical properties and hemodynamics during HPPH-mediated photodynamic therapy of pleural malignancies

**DOI:** 10.1117/1.JBO.27.10.105006

**Published:** 2022-10-31

**Authors:** Ryan D. Hall Morales, Yi Hong Ong, Jarod Finlay, Andreea Dimofte, Charles B. Simone, Joseph S. Friedberg, Theresa M. Busch, Keith A. Cengel, Timothy C. Zhu

**Affiliations:** a University of Pennsylvania, Department of Radiation Oncology, Philadelphia, Pennsylvania, United States; b New York Proton Center, New York, United States; c Temple University Health System, Department of Surgery, Philadelphia, Pennsylvania, United States

**Keywords:** photodynamic therapy, 2-[1-hexyloxyethyl]-2-devinyl pyropheophorbide-a, diffuse reflectance, tissue optical properties

## Abstract

**Significance:**

Dosimetry for photodynamic therapy is dependent on multiple parameters. Critically, *in vivo* tissue optical properties and hemodynamics must be determined carefully to calculate the total delivered light dose.

**Aim:**

Spectroscopic analysis of diffuse reflectance measurements of tissues taken during a clinical trial of 2-(1-hexyloxyethyl)-2-devinyl pyropheophorbide-a-mediated photodynamic therapy for pleural malignancies.

**Approach:**

Diffuse reflectance measurements were taken immediately before and after photodynamic therapy. Measurements were analyzed with a nonlinearly constrained multiwavelength, multi-distance algorithm to extract tissue optical properties, tissue oxygen saturation, ${\mathrm{StO}}_{2}$, and total hemoglobin concentration (THC).

**Results:**

A total of 25 patients were measured, 23 of which produced reliable fits for optical property extraction. For all tissue types, ${\mathrm{StO}}_{2}$ ranged through [24, 100]% and [22, 97]% for pre-photodynamic therapy (PDT) and post-PDT conditions, respectively. Mean THC ranged through [69,152]  μM and [48,111]  μM, for pre-PDT and post-PDT, respectively. Absorption coefficients, $\mu_{a}$, ranged through $[0.024,3.5]\;{\mathrm{cm}}^{-1}$ and $[0.039,3]\;{\mathrm{cm}}^{-1}$ for pre-PDT and post-PDT conditions, respectively. Reduced scattering coefficients, $\mu_{s}^{\prime }$, ranged through $[1.4,73.4]\;{\mathrm{cm}}^{-1}$ and $[1.2,64]\;{\mathrm{cm}}^{-1}$ for pre-PDT and post-PDT conditions, respectively.

**Conclusions:**

There were similar pre- and post-PDT tissue optical properties and hemodynamics. The high variability in each parameter for all tissue types emphasizes the importance of these measurements for accurate PDT dosimetry.

## 1 Introduction

Photodynamic therapy (PDT) is a two-stage light-activated drug therapy for the treatment of various diseases, such as oral, bladder, brain, obstructing esophageal, micro-invasive lung cancers, and many other premalignant and nononcologic diseases.^1^^–^^3^ The nature of PDT allows it to be a highly localized treatment, which is advantageous for reducing normal tissue damage and systemic toxicity. However, PDT is a complex process that depends on parameters (e.g., light fluence and propagation, local photosensitizer (PS) drug concentration, local blood flow, and oxygen concentration) that may vary spatially and temporally. Due to the highly dynamic nature of PDT, accurate dose quantification has proven to be a significant challenge.^3^^,^^4^ Since light propagation depends on the medium’s optical properties, measurement of tissue optical properties is of utmost importance for accurate quantification of PDT efficacy.^5^ Additionally, tissue optical properties can undergo significant changes following the delivery of PDT, though there is no clear trend of how they change post-PDT.^6^^–^^8^ Treatment light fluence, local drug concentration, and blood flow can be measured using fiber-based detectors coupled to the appropriate spectroscopic setup. *In vivo* light fluence distribution can be measured by means of isotropic detectors,^9^ drug distribution, and tissue oxygenation can be measured by absorption, fluorescence, and reflectance spectroscopy.^10^^,^^11^ This paper reports an update on a completed phase 1 clinical trial (NCT01673074) at the University of Pennsylvania Hospital that studied the use of 2-[1-hexyloxyethyl]-2-devinyl pyropheophorbide-a (HPPH)-mediated PDT for pleural malignancies. Using *in vivo* diffuse reflectance spectroscopy by means of a custom fiber-based contact probe, we assess relevant tissue optical properties intra-operatively. We report $\mu_{a}$ and $\mu_{s}^{\prime }$, the absorption and reduced scattering coefficients, of accessible patient organs and pleural tissue obtained immediately prior and following PDT. Additionally, we report derived effective attenuation coefficients, $\mu_{\mathrm{eff}}$, tissue oxygen saturation, ${\mathrm{StO}}_{2}$, and total hemoglobin concentration, THC, under the same conditions.

## 2 Methods and Materials

### 2.1 Patient Treatment Protocol

A total of 39 patients [age ranges 32–82 years, 75% (25%) male (female)] were enrolled in the phase 1 clinical trial (NCT01673074, informed consent was obtained for all patients that participated) performed at the University of Pennsylvania Hospital. The purpose of this trial was to study the use of HPPH-mediated PDT for treatment of pleural malignancies following bulk tumor resection. Patients were administered $4\;\mathrm{mg}/m^{2}$ (∼0.1  mg/kg) HPPH intravenously 48 h prior to surgery (this time interval is also known as the “drug-light” interval). Once tumor was resected down to <0.5  cm by the surgeon, the pleural cavity was filled with a lipoprotein colloidal suspension, 0.01% Intralipid (Fresenius Kabi, Uppsala, Sweden), to assist with uniform light scattering throughout the pleural cavity. Light therapy was administered at 665 nm to a total fluence escalation scheme of 15 to $45\;J/{\mathrm{cm}}^{2}$ with the use of a diffusing fiber optical probe. The light delivery probe has been explained previously.^12^ Briefly, it consisted of an optical fiber within an endotracheal tube with its balloon cuff filled with Intralipid scattering solution. Total light fluence was monitored at eight locations within the pleural cavity by means of fiber-based isotropic detectors. One of the detectors simultaneously monitored HPPH fluorescence. Light delivery was performed by the radiation oncologist until the prescribed dose was achieved at all locations. Diffuse reflectance and fluorescence measurements of various organs and tissue accessible within the pleural cavity were taken prior to and after light therapy (no Intralipid scattering solution was present within the pleural cavity during measurements) with a custom-made contact probe based on a previous design by Wilson et al.^4^ Surgical lights were dimmed and filtered throughout the entire treatment process. Of the 39 enrolled patients, absorption spectroscopy was performed on 25 patients. Of those patients, 23 produced adequate spectral fits for which optical properties could be reliably extracted. No fluorescence analysis was performed in this study.

### 2.2 *In Vivo* Diffuse Reflectance Spectroscopy Measurements

Spectroscopic measurements were performed on treated tissue immediately prior to and after the full treatment dose was administered. The probe used for these measurements has been discussed thoroughly in previous works.^6^^,^^10^^,^^13^^,^^14^ In short, the basic structure consisted of two 365-μm diameter optical fibers to deliver light from a quartz tungsten halogen lamp (Avalight, Avantes, Inc.), used to perform reflectance measurements. The light source fiber was coupled directly with the tissue being measured. Detector fibers located at varying source-detector (S-D) distances (0.34 to 7.8 mm) were coupled to a spectrograph and charge-coupled device (CCD) camera (InSpectrum, Roper Scientific, Princeton, New Jersey) for full spectral measurements. Spectroscopic imaging sequencing was performed as diffuse reflectance measurements, background (both sources off), and excitation fluorescence measurements. The sequence was controlled by in-house software to allow rapid acquisition and event logging, reducing the amount of user error. To reduce spectroscopic noise, the average of six measurement cycles is used as the final measurement.

### 2.3 Diffuse Reflectance Spectroscopy Data Analysis

Light propagation through homogeneous turbid media has been modeled previously by well-known solutions to the diffusion equation. ^15^^,^^16^ This solution characterizes tissue with a semi-infinite geometry by use of steady-state diffuse reflectance with the hybrid diffusion-P3 approximation.^10^^,^^17^ Expanding on this work from Hull and Foster, extracting optical properties from the obtained diffuse reflectance signal has been achieved by Finlay and Foster.^14^^,^^18^ This solution is typically employed as a wavelength-wise fit algorithm. However, the analysis in this paper uses a hybrid-P3 approximation^18^ fitting approach that can more accurately fit the diffuse approximation in semi-infinite turbid media,^19^ so long as absorbers are accounted for. The algorithm simultaneously fits for the full spectra for all S-D distances, minimizing crosstalk between the desired optical properties. Crucially, the model depends on the assumption that absorption in the medium is modeled as a linear combination of absorption of known chromophores present in the medium with positive concentrations,^20^ i.e.,

$$\mu_{a}(\lambda )=\underset{i}{\sum }\varepsilon_{i}(\lambda )c_{i},$$ (1)

where $c_{i}$ and $\varepsilon_{i}$ are the concentration and absorption spectra of the i’th absorber. In this study, we accounted for absorbance from water, hemoglobin, deoxyhemoglobin, scattering, and HPPH by use of pre-measured absorption basis spectra that is used in singular value decomposition (SVD) techniques.^13^ The extrapolated HPPH concentrations were not reliable (many null concentrations from inadequate fits), so we decided to not show the results in the paper. An exponentially weighted Fourier component series was also included to account for unknown components. Finally, we constrain the form of $\mu_{s}^{\prime }$ to be a generic form that approximates tissue attenuation well enough for our purposes:

$$\mu_{s}^{\prime }(\lambda )=A{\left(\frac{\lambda }{\lambda_{0}}\right)}^{-b},$$ (2)

with A and b free parameters returned by our fit. Since these fits produce average values of tissue deoxyhemoglobin and oxyhemoglobin concentration, we determined average tissue hemoglobin oxygen saturation (${\mathrm{StO}}_{2}$) and total hemoglobin concentration (THC), for all viable measurement samples.

### 2.4 Diffuse Reflectance Analysis and Tissue Optical Property Determination

**Fig. 1:**
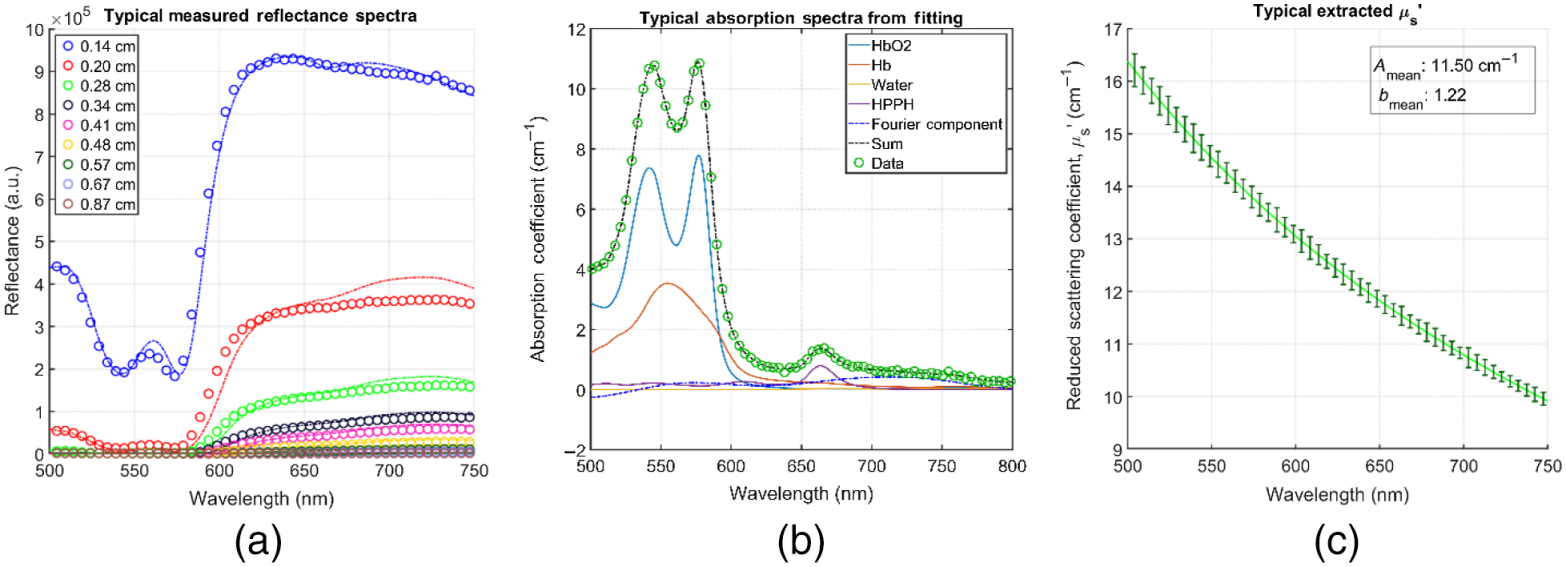
(a) Typical raw reflectance data for all S-D distances and their resulting fits for a post-PDT tumor sample; (b) a typical resulting component absorption spectrum obtained through SVD; and (c) a typical extracted scattering fit of the form $\mu_{s}^{\prime }=A{\left(\lambda /\lambda_{0}\right)}^{-b}$. (b) The total spectra (sum of each component), 61-term Fourier component that accounts for unknown absorbers, and raw data are also included. The fit-obtained component concentrations for this sample were: 65.74, 33.9, 1.15, and 3.32  μM for ${\mathrm{HbO}}_{2}$, Hb, water, and HPPH, respectively. (c) The curve is the mean of all S-D distances, and the error bars are the standard error every five data points).

Two graphical user interfaces originally developed by Finlay et al.^13^^,^^14^ in MATLAB R2015 (The MathWorks, Incorporated, Natick, Massachusetts) containing a full, modular data processing and fitting package were used in this study. Using this package, we first processed raw data from the spectrograph, applying necessary corrections [namely, background room light subtraction, $R_{\mathrm{bg}}$, subtracting dark frames to account for CCD offset, and lamp spectrum correction by normalizing the data to the spectra of a 6 inch uniform integrating sphere, $R_{\text{sphere}}$, thus the raw spectra, R is used to obtain the normalized spectra as $R_{\text{norm}}=(R-R_{\mathrm{bg}})/(R-R_{\text{sphere}})$] in the process. Once the raw data is corrected and processed, we performed the spectral fitting to extract our desired optical properties. The spectral fitting was performed in the 500- to 800-nm range in 0.49-nm intervals using a nonlinearly constrained optimization function offered in MATLAB, fmincon.m. Optical properties were extracted at the relevant wavelength for this treatment, 665 nm (the HPPH characteristic wavelength). Verification of wavelength conversion was also performed. An average of five frames was processed as the final data (discarding the first frame of every sample due to inadequate signal acquisition). The average of all nine S-D distance values obtained was used as our result. Figure 1 shows an example of raw diffuse reflectance spectral data for all S-D distances [Fig. 1(a)], a typical spectral component decomposition for a post-PDT tumor sample in the 500- to 800-nm range with 0.49-nm wavelength intervals [Fig. 1(b)], and a typical extracted scattering fit of the form $\mu_{s}^{\prime }=A{\left(\lambda /\lambda_{0}\right)}^{-b}$ [Fig. 1(c)]. The sum of each component contribution, (i.e., the resulting full absorption spectrum) is shown as well in Fig. 1(b).

## 3 Results

**Fig. 2:**
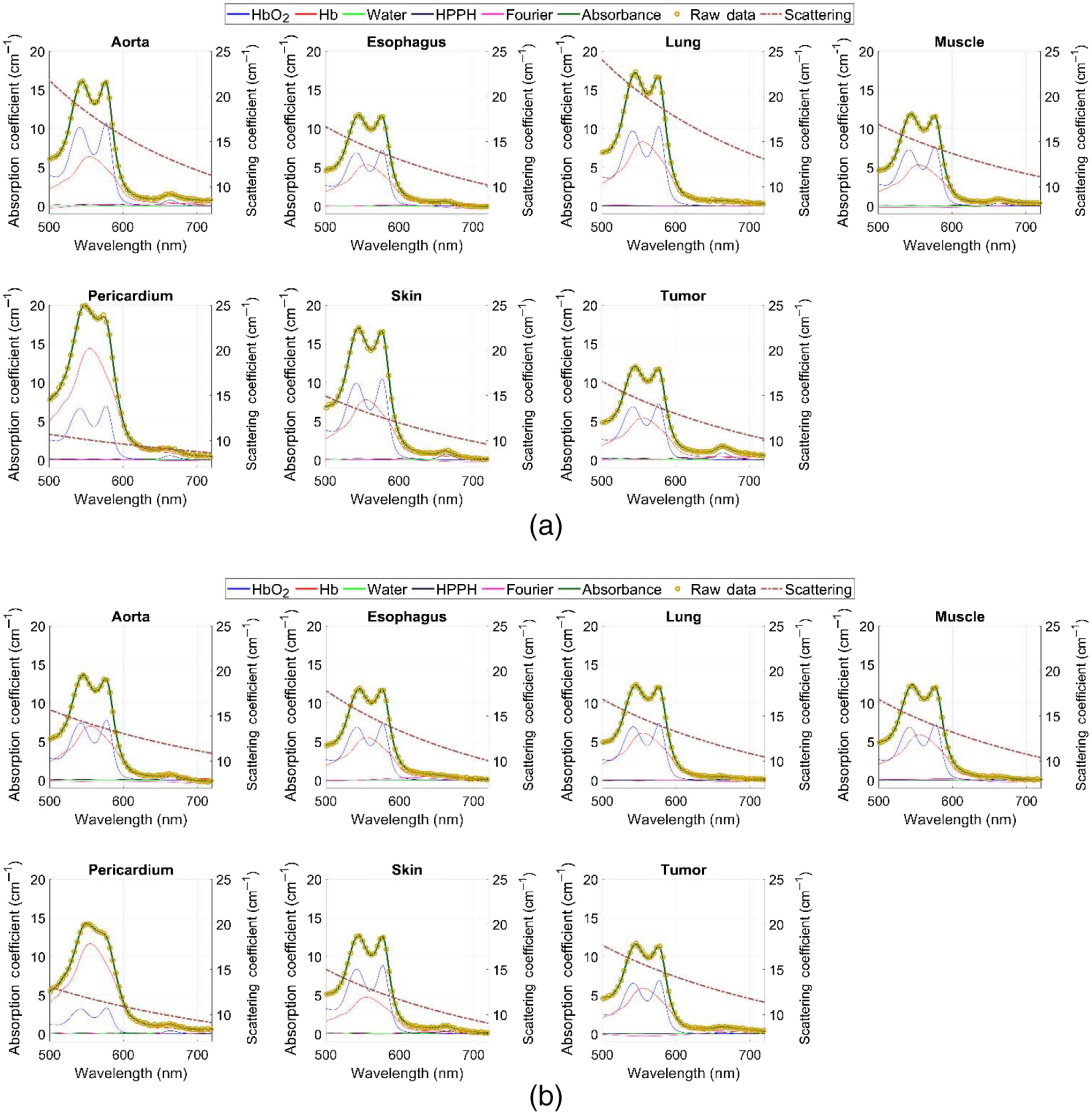
Typical total reflectance spectra of components, fit, and raw data measured from various tissue types, tumor tissue, and pleural sites during (a) pre-PDT and (b) post-PDT conditions. Absorbance raw data are yellow circular markers. Solid lines correspond to components included in analysis (including Fourier components) and fit. Each component and the fit are individually colored. Additionally, mean fit scattering spectra is included as the dashed line, with the right axis corresponding to the reduced scattering coefficient ($\mu_{s}^{\prime }$) values.

**Fig. 3:**
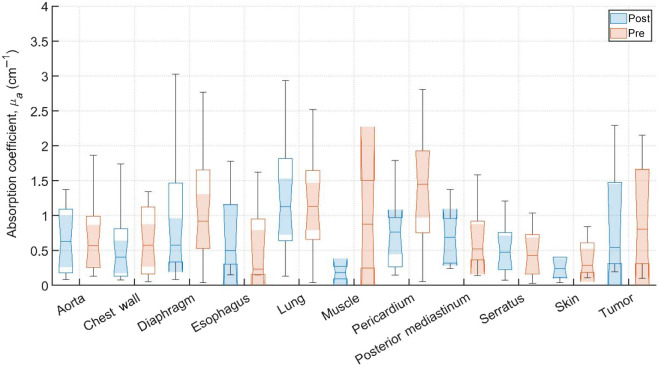
Absorption coefficient, $\mu_{a}$ (${\mathrm{cm}}^{-1}$), for various tissue types, tumor tissue, and pleural sites. The results are expressed as median values (center horizontal lines within boxes), the 95% CI (shaded notch region), IQR (entire box), non-outlier (defined as values contained within the 1.5×IQR) maximum and minimum values (horizontal whiskers), for pre-PDT and post-PDT conditions at 665 nm, obtained from diffuse spectral measurement analysis.

**Fig. 4:**
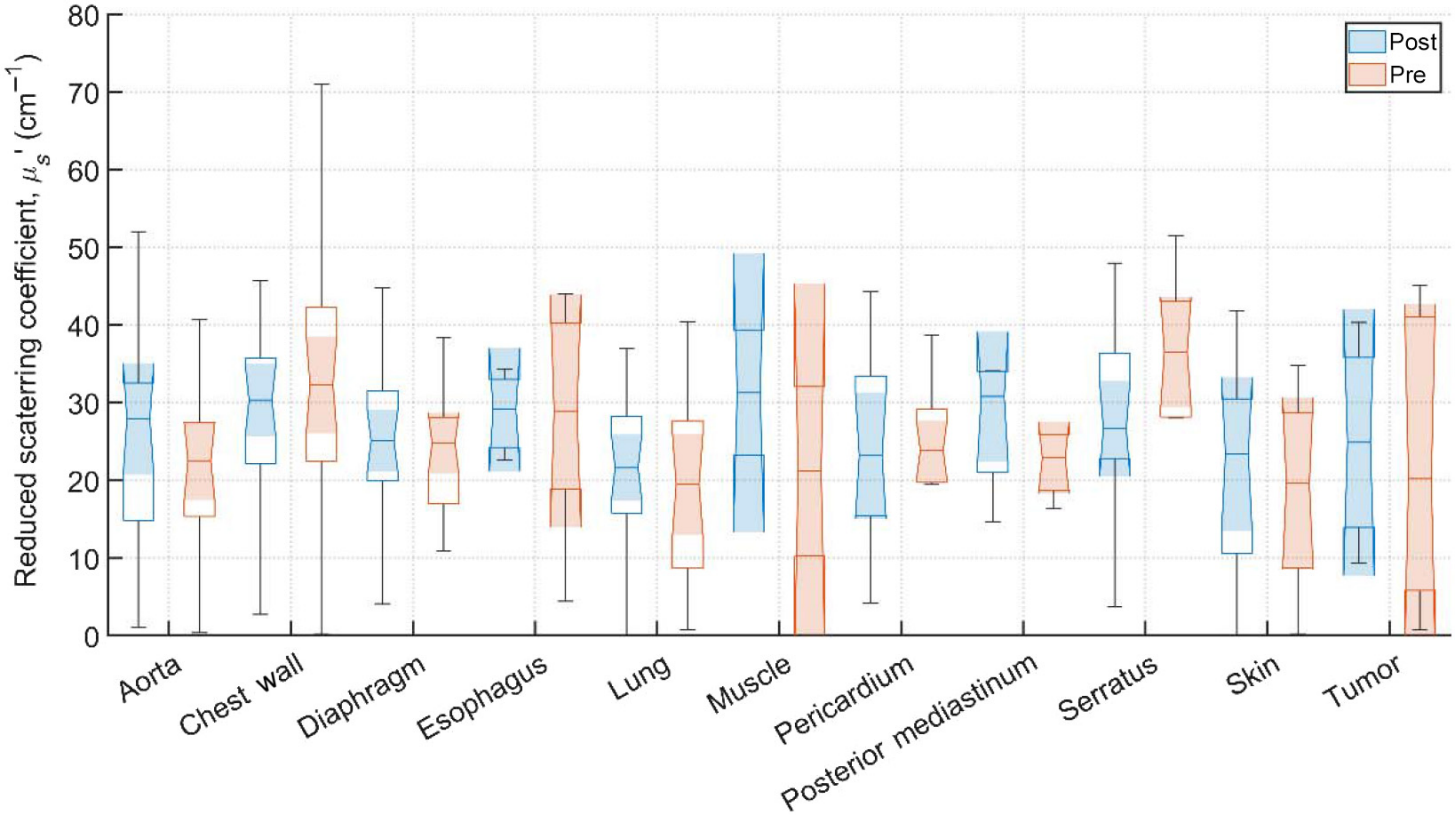
Reduced scattering coefficients, $\mu_{s}^{\prime }$, (${\mathrm{cm}}^{-1}$) for various tissue types, tumor tissue, and pleural sites. The results are expressed as median values (center horizontal lines within boxes), the 95% CI (shaded notch region), IQR (entire box), non-outlier (defined as values contained within the 1.5×IQR) maximum and minimum values (horizontal whiskers), for pre-PDT and post-PDT conditions at 665 nm, obtained from diffuse spectral measurement analysis.

**Fig. 5:**
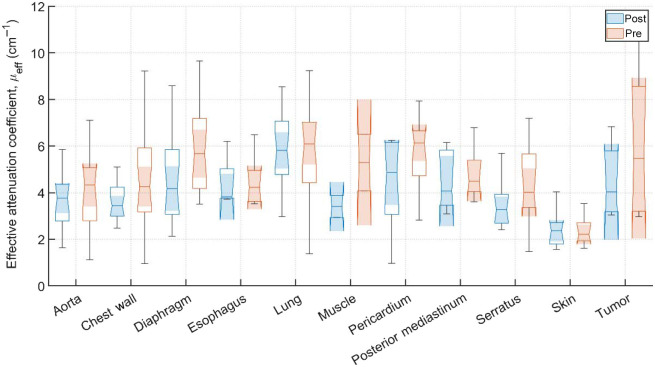
Effective attenuation coefficients, $\mu_{\mathrm{eff}}$, (${\mathrm{cm}}^{-1}$) for various tissues and pleural sites. The results are expressed as median values (center horizontal lines within boxes), the 95% CI (shaded notch region), IQR (entire box), non-outlier (defined as values contained within the 1.5×IQR) maximum and minimum values (horizontal whiskers), for pre-PDT and post-PDT conditions at 665 nm, obtained from diffuse spectral measurement analysis.

**Fig. 6:**
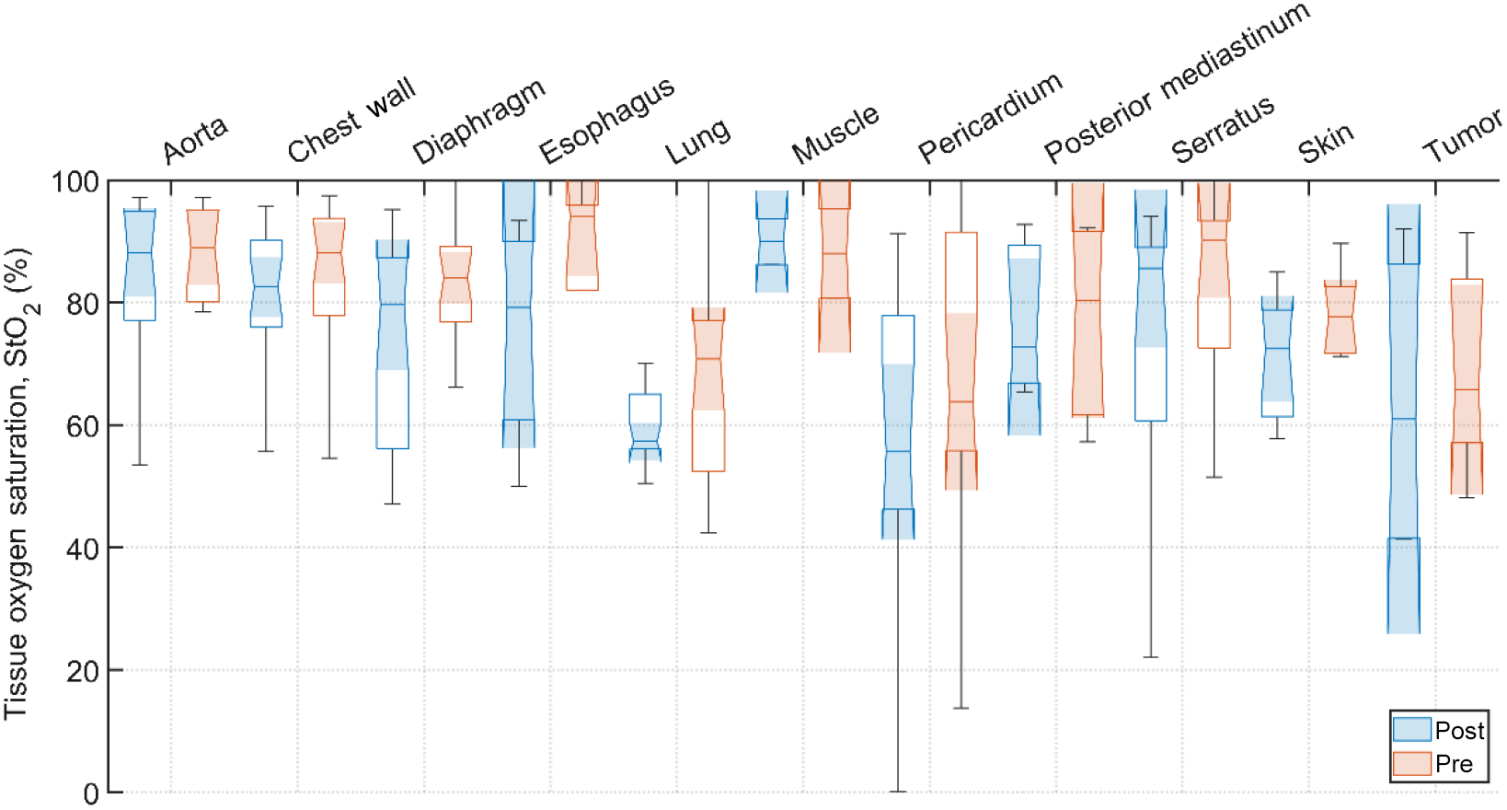
Tissue oxygen saturation, ${\mathrm{StO}}_{2}$, (%) for various tissue types, tumor tissue, and pleural sites. The results are expressed as median values (center horizontal lines within boxes), the 95% CI (shaded notch region), IQR (entire box), non-outlier (defined as values contained within the 1.5×IQR) maximum and minimum values (horizontal whiskers), for pre-PDT and post-PDT conditions at 665 nm, obtained from diffuse spectral measurement analysis.

**Fig. 7:**
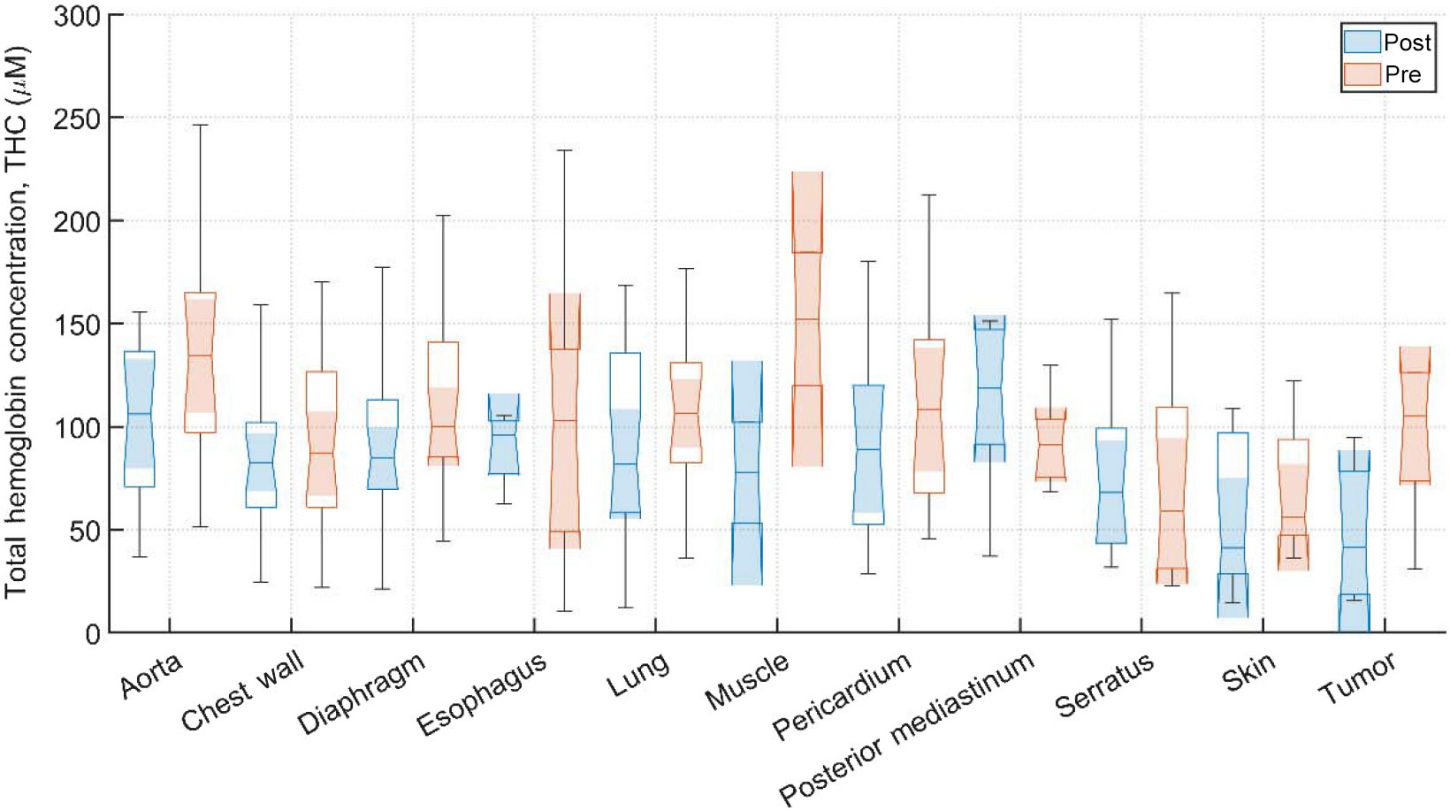
THC (μM) for various tissue types, tumor tissue, and pleural sites. The results are expressed as median values (center horizontal lines within boxes), the 95% CI (shaded notch region), IQR (entire box), non-outlier (defined as values contained within the 1.5×IQR) maximum and minimum values (horizontal whiskers), for pre-PDT and post-PDT conditions at 665 nm, obtained from diffuse spectral measurement analysis.

Typical pre-PDT to post-PDT scattering and absorption spectra and their fits for various tissue types are shown in Fig. 2. Figures 3–5 show the *in vivo* tissue optical properties, absorption coefficient $\mu_{a}$, reduced scattering coefficient $\mu_{s}^{\prime }$, and effective attenuation coefficient $\mu_{\mathrm{eff}}$, respectively, for various tissue types (aorta, esophagus, lung, muscle, pericardium, and skin), tumor tissue, and pleural sites [chest wall, diaphragm, posterior mediastinum (PM), anterior serratus A) and PS (PS and AS are lumped together)] pre- and post-PDT. Tables 1 and 2 summarize the statistics of tissue optical properties ($\mu_{a}$, $\mu_{s}^{\prime }$, and $\mu_{\mathrm{eff}}$, in ${\mathrm{cm}}^{-1}$) at 665 nm in various tissue types, tumor, and pleural sites pre- and post-PDT, respectively. Figures 6 and 7 show the oxygen saturation (${\mathrm{StO}}_{2}$, %) and THC (μM), respectively, for various tissue types (aorta, esophagus, lung, muscle, pericardium, and skin), tumor tissue, and pleural sites [chest wall, diaphragm, PM, anterior and PS (PS and AS are lumped together)] pre- and post-PDT. Tables 3 and 4 summarize the statistics of oxygen saturation (${\mathrm{StO}}_{2}$, %) and THC (μM) in various tissue types, tumor, and pleural sites pre- and post-PDT, respectively. Figures 3–7 include the 95% confidence interval (CI) as a shaded, notched reason. The figures also include the interquartile range (IQR), defined by the 25th and 75th percentiles (top and bottom of boxes). The CI and the IQR are related by $\mathrm{CI}=\text{median}\pm 1.57\times \mathrm{IQR}/\sqrt{n}$, where n is the sample size. The whiskers extending from the boxes define the non-outlier maximum and minimum values. Non-outliers were defined as values contained within 1.5×IQR. For each site, post- and pre-PDT mean optical property ($\mu_{a}$, $\mu_{s}^{\prime }$, and $\mu_{\mathrm{eff}}$) median values do not differ within a 5% significance level. The same applies for ${\mathrm{StO}}_{2}$ and THC, with the notable exception of lung ${\mathrm{StO}}_{2}$, for which pre- and post-PDT medians were 70.8% (CI: 62.4% to 79.2%) and 57.3% (CI: 54.27% to 60.41%), respectively. It is worthy to note that for many of the sites (e.g., Fig. 3 for the esophagus, muscle, and tumor), the CI extended beyond the IQR due to the uncertainty of the true median value (likely due to the low sample size for those sites).

**Table 1:** Tissue optical properties ($\mu_{a}$, $\mu_{s}^{\prime }$ and $\mu_{\mathrm{eff}}$, in ${\mathrm{cm}}^{-1}$) at 665 nm under pre-PDT conditions for various tissues and pleural sites. The results are expressed as mean (SD). Sample counts are the number of samples taken for each site across all successfully analyzed patients.

Tissue	Sample count	$\mu_{a}$ (${\mathrm{cm}}^{-1}$)	$\mu_{s}^{\prime }$ (${\mathrm{cm}}^{-1}$)	$\mu_{\mathrm{eff}}$ (${\mathrm{cm}}^{-1}$)
Aorta	15	0.7 (0.5)	21 (13)	4.2 (2.1)
Chest wall	25	0.8 (0.8)	32 (18)	4.7 (2.3)
Diaphragm	21	1.2 (0.9)	25 (9)	5.8 (1.9)
Esophagus	5	0.6 (0.6)	28 (15)	4.5 (1.2)
Lung	21	1.3 (0.9)	20 (12)	5.8 (1.9)
Muscle	2	0.9 (0.9)	21 (15)	5.3 (1.7)
Pericardium	15	1.3 (0.8)	23 (13)	5.8 (1.4)
PM	6	0.7 (0.5)	24 (7)	4.8 (1.1)
Serratus	12	0.5 (0.5)	32 (17)	4.3 (1.7)
Skin	8	0.5 (0.5)	19 (13)	2.4 (0.6)
Tumor	6	1.0 (0.8)	22 (18)	6.1 (3.0)

**Table 2:** Tissue optical properties ($\mu_{a}$, $\mu_{s}^{\prime }$ and $\mu_{\mathrm{eff}}$, in ${\mathrm{cm}}^{-1}$) at 665 nm under post-PDT conditions for various tissues and pleural sites. The results are expressed as: mean (SD). Sample counts are the number of samples taken for each site across all successfully analyzed patients.

Tissue	Sample count	$\mu_{a}$ (${\mathrm{cm}}^{-1}$)	$\mu_{s}^{\prime }$ (${\mathrm{cm}}^{-1}$)	$\mu_{\mathrm{eff}}$ (${\mathrm{cm}}^{-1}$)
Aorta	15	0.6 (0.5)	26 (15)	3.7 (1.2)
Chest wall	21	0.5 (0.6)	29 (14)	3.8 (1.2)
Diaphragm	21	0.9 (0.8)	27 (14)	4.6 (1.8)
Esophagus	4	0.7 (0.7)	29 (6)	4.4 (1.2)
Lung	21	1.2 (0.7)	21 (12)	5.8 (1.6)
Muscle	2	0.2 (0.1)	31 (11)	3.4 (0.7)
Pericardium	12	0.7 (0.5)	24 (13)	4.5 (1.7)
PM	6	0.7 (0.4)	28 (8)	4.4 (1.3)
Serratus	12	0.5 (0.4)	29 (12)	3.5 (1.1)
Skin	10	0.3 (0.3)	23 (19)	2.4 (0.7)
Tumor	4	0.9 (0.9)	25 (4)	4.5 (1.7)

**Table 3:** Summary of tissue oxygen saturation values (%) under pre-PDT conditions for various tissues and pleural sites. The results are expressed as mean (SD). Sample counts are the number of samples taken for each site across all successfully analyzed patients.

Tissue	Sample count	$S_{t}O_{2}$ (%)	THC (μM)
Aorta	15	84 (17)	135 (58)
Chest wall	25	83 (16)	94 (42)
Diaphragm	21	82 (11)	115 (43)
Esophagus	5	86 (21)	103 (82)
Lung	21	68 (16)	113 (52)
Muscle	2	88 (10)	152 (45)
Pericardium	15	67 (26)	109 (47)
PM	6	77 (16)	93 (22)
Serratus	12	83 (15)	74 (51)
Skin	8	76 (11)	69 (33)
Tumor	6	69 (17)	69 (17)

**Table 4:** Summary of tissue oxygen saturation values (%) under post-PDT conditions for various tissues and pleural sites. The results are expressed as mean (SD). Sample counts are the number of samples taken for each site across all successfully analyzed patients.

Tissue	Sample count	$S_{t}O_{2}$ (%)	THC (μM)
Aorta	15	84 (14)	102 (38)
Chest wall	21	77 (21)	85 (37)
Diaphragm	21	74 (16)	93 (36)
Esophagus	4	75 (19)	90 (19)
Lung	21	60 (16)	92 (47)
Muscle	2	90 (5)	77 (34)
Pericardium	12	58 (25)	92 (47)
PM	6	77 (12)	110 (43)
Serratus	12	74 (22)	75 (38)
Skin	10	66 (23)	56 (37)
Tumor	4	64 (26)	48 (37)

## 4 Discussion

### 4.1 Tissue Optical Properties

A total of 39 patients were treated with HPPH-mediated PDT for pleural malignancies. Due to the complexity of the treatment logistics, 25 patients were evaluated for spectroscopic tissue analysis. The measurements from five patients were discarded due to measurement instabilities and poor quality fits to reflectance spectra which resulted in large uncertainties in the extracted tissue optical properties. The reliability of extracted HPPH concentration was affected most primarily due to the relatively short-lived and inherently weak HPPH signal, as well as other factors such as inconsistent pressure applied on the probe and uncertainties in the fitting algorithm, etc. Of the 20 successfully analyzed patients, only two produced adequate component fits that exhibited obvious contributions from the presence of HPPH. Components relating to the presence of oxy- and deoxy-hemoglobin (${\mathrm{StO}}_{2}$ and THC), however, can still be reliably extracted as the contribution of HPPH absorption to the entire reflectance spectra is relatively small. These components represent the bulk contribution to tissue optical properties, and thus $\mu_{a}$, $\mu_{s}^{\prime }$, and $\mu_{\mathrm{eff}}$ can be reliably extracted for all analyzed patients presented.

Measurements on the remaining patients were performed in varying locations, depending on tissue accessibility during treatment. The data was then processed, applying the previously mentioned spectral corrections that account for surgical light background, CCD offset, and tungsten lamp spectrum. Fitting was performed with a multiwavelength spectral fitting algorithm that simultaneously fits for all S-D distances, centered at 665 nm. The average of all nine S-D optical property values was averaged for our result that tissue. All sample counts, mean and standard deviations (SDs) for all coefficient values under pre-PDT and post-PDT conditions can be found in Tables 1 and 2, respectively. Additionally, Figs. 3, 4, and 5 show the median, upper and lower quartile ranges, non-outlier maximum and minimum values, as well as outliers (calculated using the interquartile range) for pre-PDT and post-PDT $\mu_{a}$, $\mu_{s}^{\prime }$, and $\mu_{\mathrm{eff}}$, respectively. As has been reported in previous studies,^6^ optical properties do not differ with any meaningful pattern, particularly for $\mu_{\mathrm{eff}}$. Of note, it’s difficult to draw any meaningful conclusion for muscle tissue optical property changes due to the limited sample size (two measurements for both pre-PDT and post-PDT conditions; see Tables 1 and 2).

A comparison of tissue optical properties between HPPH- and Photofrin-mediated pleural PDT can be made. Based on the mean tissue optical properties for HPPH to be $\mu_{a}=0.92\pm 0.79\;{\mathrm{cm}}^{-1}$ and $\mu_{s}^{\prime }=24.3\pm 14.7\;{\mathrm{cm}}^{-1}$, thus $\mu_{\mathrm{eff}}=8.2\;{\mathrm{cm}}^{-1}$ and that for Photofrin to be $\mu_{a}=0.37\pm 0.15\;{\mathrm{cm}}^{-1}$ and $\mu_{s}^{\prime }=9.4\pm 2.2\;{\mathrm{cm}}^{-1}$, thus $\mu_{\mathrm{eff}}=3.2\;{\mathrm{cm}}^{-1}$.^11^ We concluded that the light penetration depth may be shorter for HPPH ($1/\mu_{\mathrm{eff}}=0.12\;\mathrm{cm}$) than that for Photofrin ($1/\mu_{\mathrm{eff}}=0.31\;\mathrm{cm}$). The result is surprising since the optical penetration depth generally increases with increasing wavelengths.

### 4.2 Tissue Oxygen Saturation, ${\mathrm{StO}}_{2}$

All sample counts, mean, and SD for all tissue ${\mathrm{StO}}_{2}$ values under pre-PDT and post-PDT conditions can be found in Tables 3 and 4, respectively. Additionally, Fig. 6 shows the median, upper and lower quartile ranges, non-outlier maximum and minimum values, as well as outliers (calculated using the interquartile range) for pre-PDT and post-PDT tissue ${\mathrm{StO}}_{2}$. In the case of ${\mathrm{StO}}_{2}$, mean values were (78±17)% before PDT and were (71±21)% after PDT. Thus, ${\mathrm{StO}}_{2}$ dropped for most tissues, except the aorta, PM, and muscle (again, only two pre-PDT and post-PDT samples each were analyzed). However, the relative differences in all but three tissues were <10% reduction, producing meaningful difference in tissue oxyhemodynamics post-treatment.

### 4.3 Total Hemoglobin Concentration

All sample counts, mean and SDs for all tissue THC values under pre-PDT and post-PDT conditions can be found in Tables 3 and 4, respectively. Additionally, Fig. 7 shows the median, upper and lower quartile ranges, non-outlier maximum and minimum values, as well as outliers (calculated using the interquartile range) for pre-PDT and post-PDT tissue THC. Mean values of THC were (108±54)  μM before PDT and were (85±41)  μM after PDT. Thus, for all tissues except the Serratus, THC mean values decreased from pre-PDT to post-PDT conditions. However, mean post-PDT values are well within one SD of pre-PDT values.

### 4.4 Light Penetration in Tissue

**Fig. 8:**
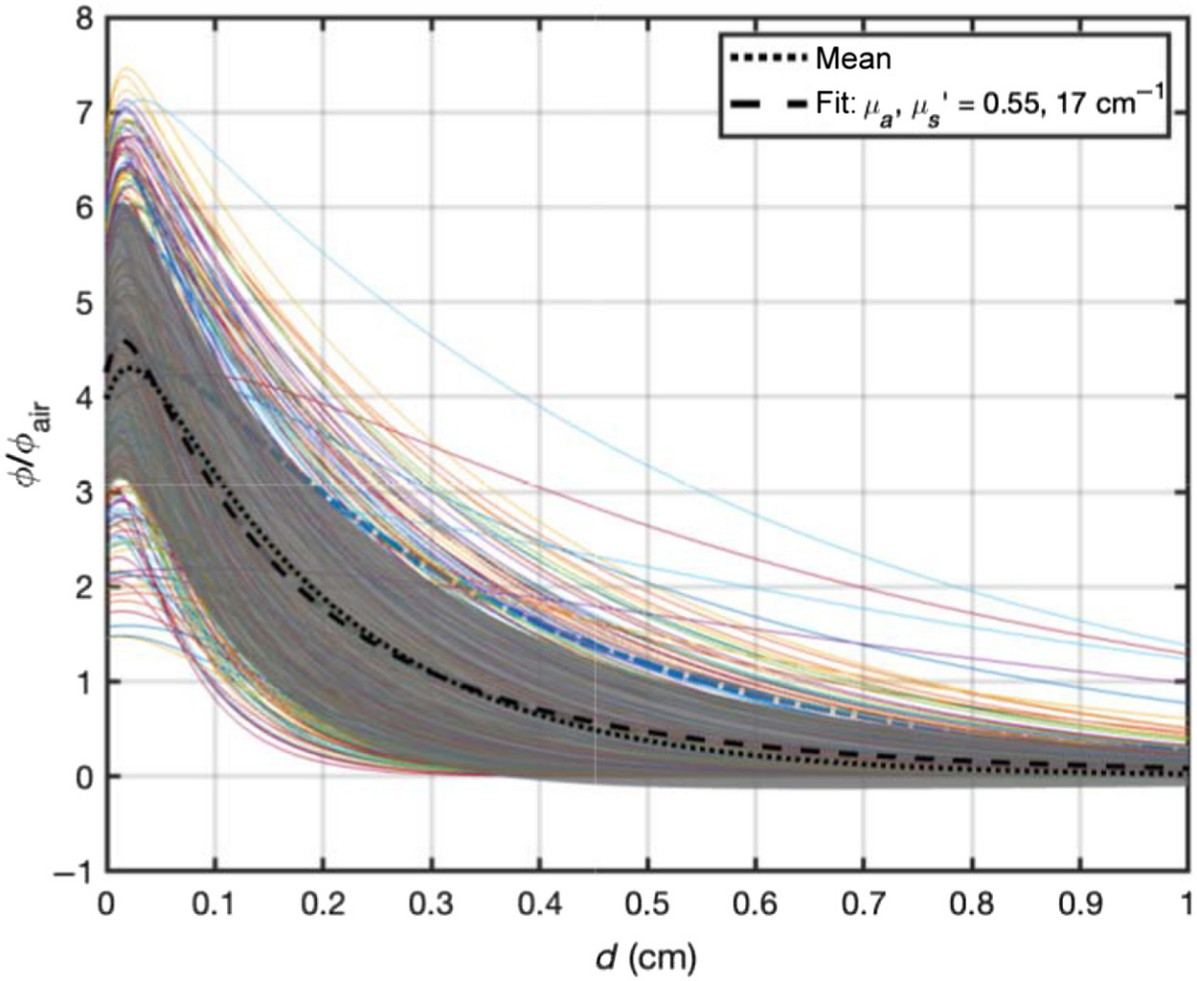
Expected light fluence rate distribution for HPPH-mediated PDT based on measured tissue optical properties before and after PDT for all tissue types. The solid lines are for each measured tissue optical properties, The dotted line is the mean of all calculated light fluence rate, the dashed line is the best fit to the mean of all light fluence rate distribution along with the resulting mean tissue optical properties: $\mu_{a}=0.55\pm 0.04\;{\mathrm{cm}}^{-1}$ and $\mu_{s}^{\prime }=17\pm 1\;{\mathrm{cm}}^{-1}$. The gray area represents one SD of the mean.

Based on the measured optical properties, one can evaluate the light penetration depth based on an analytical function for broad beam light incident on a semi-infinite surface.^21^ Figure 8 shows that the ratio of light fluence rate to in-air fluence rate, defined as the calculated incident fluence rate on the turbid medium surface (S/A, where S is the laser power and A is the area of illumination), as a function of tissue depth (in cm). The solid lines represent the light fluence rate distribution versus depth for measured tissue optical properties. The dotted line represents the mean of all light fluence rate distribution and the grey area represents 1 SD of the mean. The dashed line is best fit to the mean curve using the same analytical function for broad beam incident on a semi-infinite medium and the resulting mean tissue optical properties ($\mu_{a}=0.55\pm 0.04\;{\mathrm{cm}}^{-1}$ and $\mu_{s}^{\prime }=17\pm 1\;{\mathrm{cm}}^{-1}$). Thus, the effective penetration depth is $1/\mu_{\mathrm{eff}}=0.18\;\mathrm{cm}$, however, the light can penetrate to a depth of 0.30 cm ($\phi /\phi_{\text{air}}=1$, dashed line) because of the increased light fluence rate near surface due to tissue backscattering. At a depth of 0.5 cm, the mean light fluence rate is expected to be $0.37\phi_{\text{air}}$.

This paper summarizes results for the tissue optical property and spectroscopic analysis for the HPPH-mediated pleural PDT clinical trial (NCT01673074). We see similar optical properties pre- and post-PDT. Furthermore, this analysis supports the pattern from similar PDT spectroscopic studies for different PSs that show tissue optical properties to be highly heterogeneous between inter-patient and intra-patient tissue samples for both canine and human patients.^6^^,^^13^^,^^14^ This is to be expected due to the inherent heterogeneity of tissue in general. Tissue oxygenation plays a critical role in PDT dose delivery;^8^^,^^10^^–^^12^ therefore, its accurate measurement plays a significant role in PDT dosimetry. This further supports the conclusion that accurate PDT dosimetry requires not only accurate quantification of light fluence, drug concentration, and tissue oxygenation, but also accurate spectroscopic tissue analysis. It would be insufficiently accurate to perform dosimetry using standard tissue optical properties data sets. Rather, spectroscopic analysis should be performed for all tissue locations of concern before and/or after treatment for every patient.

## Code, Data, and Materials Availability

The anonymized patient data and code utilized for data analysis and generation of all results are available upon request.
